# Network spectra for drug-target identification in complex diseases: new guns against old foes

**DOI:** 10.1007/s41109-018-0107-y

**Published:** 2018-12-17

**Authors:** Aparna Rai, Pramod Shinde, Sarika Jalan

**Affiliations:** 1 Aushadhi Open Innovation Programme, Indian Institute of Technology Guwahati, Guwahati, 781039 India; 2 Discipline of Biosciences and Biomedical Engineering, Indian Institute of Technology Indore, Khandwa Road, Simrol, Indore, 453552 India; 3 Complex Systems Lab, Discipline of Physics, Indian Institute of Technology Indore, Khandwa Road, Indore, 453552 India; 4 Lobachevsky University, Gagarin avenue 23, Nizhny Novgorod, 603950 Russia

**Keywords:** Disease networks, Network spectra, Biomarkers, Random matrix theory (RMT), Systems biology

## Abstract

The fundamental understanding of altered complex molecular interactions in a diseased condition is the key to its cure. The overall functioning of these molecules is kind of jugglers play in the cell orchestra and to anticipate these relationships among the molecules is one of the greatest challenges in modern biology and medicine. Network science turned out to be providing a successful and simple platform to understand complex interactions among healthy and diseased tissues. Furthermore, much information about the structure and dynamics of a network is concealed in the eigenvalues of its adjacency matrix. In this review, we illustrate rapid advancements in the field of network science in combination with spectral graph theory that enables us to uncover the complexities of various diseases. Interpretations laid by network science approach have solicited insights into molecular relationships and have reported novel drug targets and biomarkers in various complex diseases.

## Background

Biomolecules in a living organism rarely act individually. Instead, they work together in a cooperative way to provide specific functions. In other words, each of the biomolecules is a set of functioning assistants to other molecules that helps in proper cellular signaling. The overall functioning of these molecules is of jugglers play in the cell orchestra (Burz and Shekhtman 2009). The functioning of the cell may take on a very different character if even a single member of this molecular orchestra starts behaving strangely (Thomas et al.). Some disease states are a consequence of one or many of such flaws in molecular interactions that eventually result in the altered dynamics of expressions of the differential molecules (Boorse 1975; Ereshefsky 2009). Understanding the relationships among these altered molecular interactions and consequently finding the change in the condition of an entire cell is one of the greatest challenges in modern biology and medicine (Alyass et al. 2015; Melnik et al. 2017; Ayers and Day P 2015; Sneha and George P 2016).

**Fig. 1:**
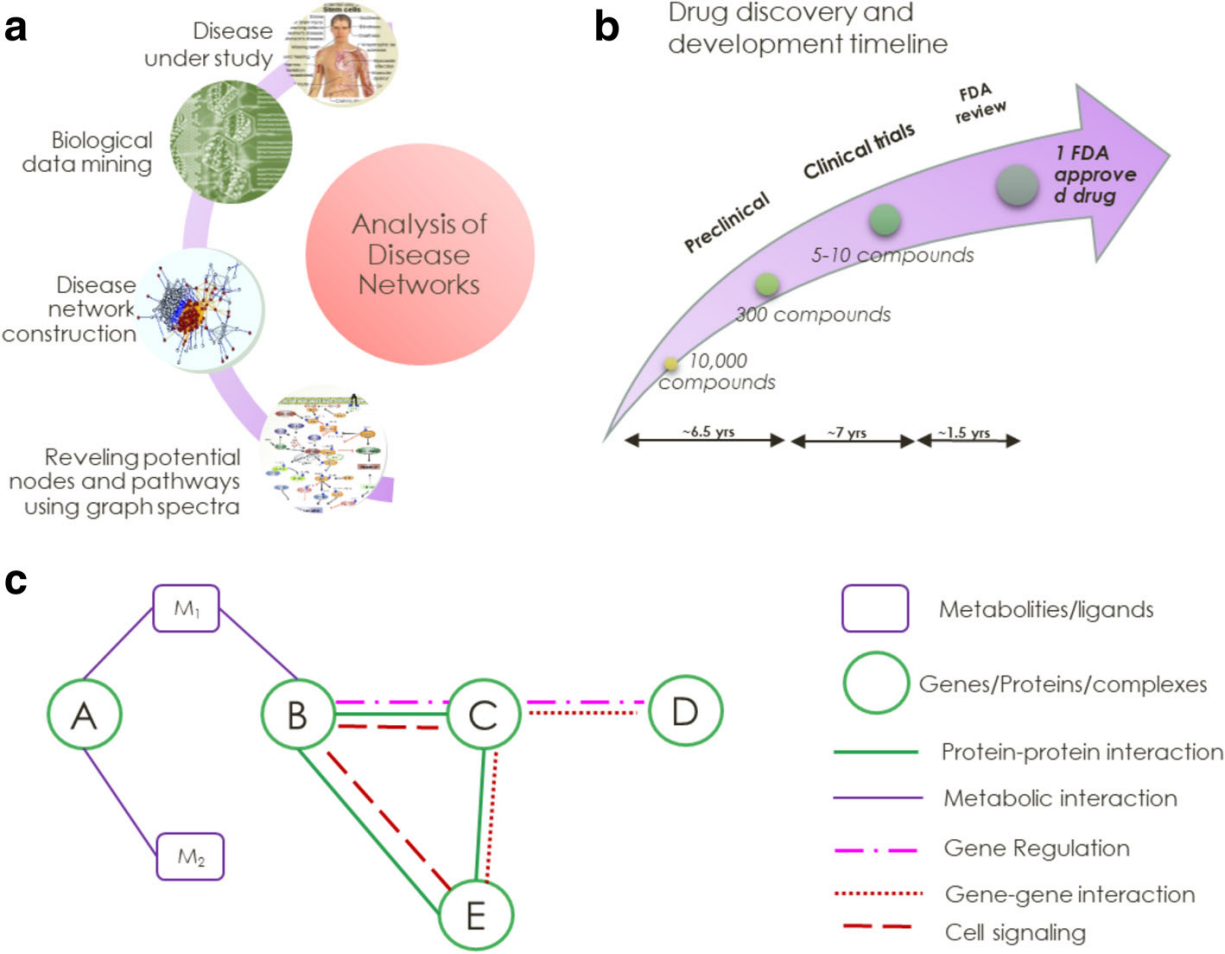
Representative diagrams. **a** Steps to identify drug targets using network spectra. Step-wise, it involves biological data mining of disease in interest, it can be any biological data such as of sequence data, expression data etc. Further, disease network is constructed using the biological data and after that various techniques in spectral graph theory are exploited to identify important information in networks. **b** Drug discovery and development timeline. It starts from target identification to pre-clinical studies, to 4 tier clinical trails. From start to finish, the entire drug development process usually spans about 8 to 12 years, leaving drug developers with around a decade or less of patent exclusivity on branded drugs once they make it to market. **c** Types of biological interactions that can be represented by networks. Molecular interactions are effects that biomolecules have on each other. Since there are variety of biomolecules present such as proteins, DNA, there are diverse types of interactions among biomolecules are possible

The post-genomic era aims to understand human health and diseases by investigating the role of macromolecules (Venter and et al. 2001). According to a recent study, a person with any complex disease such as cancer, diabetes, cardiovascular diseases etc spends on an average more than $85,000 in the treatment and its complications over entire lifetime (American Diabetes Association 1998; Gruber and et al. 1997). Trillions of dollars are spent on health and diseases including cancer, diabetes, neural diseases, etc. worldwide (Sepúlveda and Murray 2014). Much of these expenses are incurred by the pharmaceutical sector *i.e.,* in early phases of the development of (drug like) compounds. Less than 0.1% of these compounds are approved as drugs after 7-10 years of clinical trials (Fig. 1(b)). Therefore, the rates of success/failure of potential drug-like compounds are critical to the cost of drug discovery process. Lack of target specificity and inactivity of these compounds are two primary reasons for drug failure (Omudhome and Pharm 2002).

Furthermore, we are currently witnessing a resurgence of interest in use of large volume of biological data and systems biology approaches in drug discovery. Most of data screening is carried out by high throughput data collection techniques such as imaging, gene expression microarrays, or genome wide screening (Ayers and Day P 2015; Barabási and et al. 2011; Shinde et al. 2018; Gohil and et al. 2015; Hartwell and et al. 1999). Utilizing this vast information, rapid advancements has been taken place in both experimental and theoretical techniques in recent years (Chou 2006). However, heterogeneity exhibited by various diseases specifically in tissue type, expression and growth patterns and in cell division increases the complexity of the already complicated cellular pathways and functioning networks (Fisher and et al. 2013; Burrell and et al. 2013; Portela and Esteller 2010). Thus, analysis of such a diverse range of biological questions require development of novel tools to counter the diseasome at the systems level. Development of statistical tools may prove to be highly potent in addressing such complex disease models. One such promising approach is to consider the system as networks (Fig. 1(a)) (Kitano 2002; Zhu and et al. 2007; Barabási and Oltvai 2004; Rai 2017).

## Basic network nomenclature

Many diseases are caused by a combination of molecular perturbations. A complex disease is referred as a disease complexome or a diseasome in modern system biology era (Rai 2017). Networks present a simple framework to model complex systems comprising of a large number of interacting elements. The network for any biological system can be represented by nodes (vertices) which denote biomolecules and links (edges) which arise due to the intermolecular interactions (Fig. 1a). If a pair of biomolecules is known to have an interaction (physical, chemical or genetic) between them, that pair of the nodes is connected with a link. Mathematically, a network or a graph is defined as a set of **N** nodes and number of links which can be represented in terms of an adjacency matrix (**A**) as,

1 $$ A_{\text{ij}} = \left\{\begin{array}{ll} 1 ~~\text{if}\ i \sim j \\ 0 ~~ \text{otherwise} \end{array}\right.   $$

Establishing various intermolecular interactions is not trivial (Table 1). Intermolecular interactions including protein-protein, protein-nucleic acids, protein-metabolite are conceptually straightforward. Apart from that more complex functional interactions, determined using mathematical and statistical modeling, can also be considered. For example, gene co-expression network is constructed by looking for pairs of genes which show a similar expression pattern across samples.

**Table 1:** Various types of disease networks

To have a better understanding of several cellular processes in complex diseases, it is helpful to study how various components make up the system. Some of the most common types of disease networks are:
1. Protein - protein interaction (PPI) networks: PPI networks are perturbed, those of normal cells, in disease due to sequence mutations and expression changes. There are a multitude of methods to detect PPI’s both high-throughput methods (e.g. Yeast two-hybrid screening, mass spectrometry, protein microarrays) and bioinformatics (e.g. text mining, machine learning) based approaches. There have also been major efforts to curate the interactions that have been validated individually in the literature into databases. These databases are the Munich Information Center for Protein Sequence (MIPS, (Mewes and et al. 2002)), the Biomolecular Interaction Network Database (BIND, (Bader et al. 2003)), the Database of Interacting Proteins (DIP, (Xenarios and et al. 2000)), the Molecular Interaction database (MINT, (Ceol and et al. 2017)),and the protein Interaction database (IntAct, (Kerrien and et al. 2011)), Biological General Repository for Interaction Datasets (BioGRID, (Chatr-Aryamontri and et al. 2017)) and the Human Protein Reference Database (HPRD, (Keshava and et al. 2009)). Our current knowledge of the PPI’s is both incomplete and noisy.
2. Gene (transcriptional) regulatory networks: These subgroup of biological networks describe how gene expression is controlled and regulated. The regulator can be DNA, RNA, protein, ions and molecular complexes. There are various high-throughput experimental approaches were developed to study regulatory activities such as chromatin immuno-precipitation (ChIP) followed by microarrays (ChIP-chip) and ChIP followed by sequencing (ChIP-seq), synthetic genetic arrays.
3. Metabolic and biochemical networks: Metabolic networks represent the relationships among small biomolecules (metabolites) and the enzymes (proteins) to catalyze a biochemical reaction. These reactions allow an organism to grow, reproduce, respond to the environment and maintain its structure. Bioinformatics databases such as Kyoto Encyclopedia of Genes and Genomes (KEGG, (Kanehisa and Goto 2000)), BioCyc, (Caspi and et al. 2007), the Biochemical Genetic and Genomics knowledge base (BIGG, (Schellenberger et al. 2010)), and The Human Metabolome Database (HMDB, (Wishart and et al. 2012)) contain wide range of the metabolic networks.
4. Genetic interaction networks: Genetic interactions occur when mutations in two or more genes combine to generate an unexpected (undesired) phenotype. These networks represent a functional relationship between different genes, rather than physical one, essentially predicted by DNA sequences or gene expression profiling.
5. Cell signaling networks: These are formed when different cell pathways interact and are detected by a combination of experimental and computational methods. Cell signaling networks are systematically represented by two type resources *i.e.,* pathway databases (Reactome, wikiPathways etc.) and cellular signaling network databases (Signor, SigmaLink) (Croft and et al. 2010; Kelder and et al. 2011; Perfetto and et al. 2015; Fazekas and et al. 2013).
Apart from these mentioned categories of networks, there have been several efforts made to integrate information from various databases to a single place, such biological network databases are termed as meta-databases. For example, STRING (Szklarczyk and et al. 2017), BIANA (Garcia-Garcia and et al. 2010), ConsensusPathDB (Kamburov and et al. 2008), Human Integrated Protein-Protein Interaction Reference (HIPPIE, (Schaefer and et al. 2012)), International Molecular Exchange (IMEX, (Orchard and et al. 2012)), Agile Protein Interactomes DataServer (APID, (Prieto and Javier 2006)) etc.

## Why network spectra: an overview

### Network system biology and network pharmacology

Network theory has been tremendously successful in simplifying and understanding complex biological systems (Wang 2011). Previous attempts to understand various diseases through network system biology approach have revealed deep insight into complex diseases (Barabási and et al. 2011; Cho et al. 2012; Furlong 2013; Wang and et al. 2012; Draghici and et al. 2007). Few of these studies entails that various types of cancers are interlinked to each other through few pathways as well as these common pathways are found to be altered among different diseases (Goh and et al. 2007). Further analysis of centrosome (a cellular organelle) dysfunction under the network theory framework reveals the importance of highly connected proteins (hubs) as well as those proteins connected with these hub proteins (Pujana and et al. 2007; Chuang and et al. 2007). Network studies pertaining to epigenetic modifications, gene regulations, gene expressions, PPI’s provided insights into the molecular mechanisms of the disease. Additionally, these network studies helped in finding functionally important proteins as well as some of the missing pathways in cancer (Wang and et al. 2012; AlQuraishi and et al. 2014; Kar and et al. 2009; Jonsson and Bates 2006). Essentially, these network studies provided a global understanding to biological processes and protein interactions (Barabási and et al. 2011; Goh and et al. 2007; Creixell and et al. 2015; Califano 2014).

Surprisingly, despite networks representing these complex systems being so diverse, they possess universal behaviors (Sarkar and Jalan 2018; Rai 2017). The universalities captured by the structural or topological properties of the underlying networks provide fundamental insights of the underlying systems (Rai and Jalan 2015). However, the universal structural properties remain same for most of the biological networks, e.g., scale-free nature of the networks, small diameter and high clustering coefficients (Rai et al. 2014; Rai and et al. 2017; Jalan and et al. 2015; Rai et al. 2015). To have a deeper insight into potentially important cellular and molecular mechanisms between healthy and diseased tissue states, the combined approach of network theory and spectral graph theory has turned out to be relevant (Rai 2017). Analysis of random matrices of corresponding networks has shown tremendous success in distinguishing level of complexities among a wide variety of disciplines, being as diverse as the human brain, the world wide web, food-web, scientific collaborations, communications and power systems engineering to molecular and population biology (Albert and Barabási 2002; Newman 2002; Wigner 1955; Papenbrock and Weidenmüller 2007; Kwapień and DroŻdŻ 2012; Rai and Jalan 2015). In the recent years, the framework has shown its credibility in providing insights into various biological systems like gene co-expression networks, PPI networks, understanding the genetic variance among both species and diseases etc., and predicting important biomolecules which can be used as potential drug targets (Guney et al. 2016; Guney and Oliva 2014; Ideker and Roded 2008; Agrawal and et al. 2014; Gibson and et al. 2013; Blows and McGuigan 2015). Due to the successful application of this technique on other complex systems, the research community have recognized it as a promising application on disease networks as well (Aguirre-Plans and et al. 2018).

### Drug target identification through network spectra

Biological pathways typically yield both an expanded mesh and a comprehensive representation of biomolecules capable of assembling together into a broad neighborhood context. For example, any disease as a network comprises of interactions between various molecules and contain numerous components of the cell rather than independent interactions involving only few molecules (Ideker et al. 2001). Therefore, it is a more favorable approach to target a group of proteins (biomolecules), than focusing on a single druggable protein/biomolecule. At the molecular level, the group of proteins make a complex, metabolic or signaling pathway, a functional module, etc. and hence the development of drug discovery strategies to target such a group of proteins would be more appropriate than against any single protein (Aguirre-Plans and et al. 2018). Such an approach have been used by (Schoeberl and et al. 2009) to identify novel therapeutic target for cancer within the ErbB pathway. They first identified the most effective target ligands using the entire ErbB signaling pathway to control protein ErbB3 and further the protein targets-ligand binding was validated using the method of targeted monoclonal antibody (Schoeberl and et al. 2009).

Topological features such as node degree and clustering coefficient are informative in identifying important network components. In scale-free networks (Table 2), the large number of nodes have few connections, and few nodes are having a large number of connections termed as hubs. These make networks functionally robust. Therefore, identifying hub nodes and their targeted inhibition can be used to access overall network function. In PPI network of *Saccharomyces cerevisiae*, it has been shown that hub proteins manifested multi-domain protein 3D structure, and hence these hub proteins provide binding sites to many other proteins with diverse domain compositions (Ekman et al. 2006). Further, the emergence of most diseases cannot be explained by single-gene defects but involve the breakdown of the coordinated function of distinct gene groups (Bartlett and Zaikin A. 2016; Guney and Oliva 2014; Ideker and Roded 2008; Kitsak and et al. 2016). Networks with high clustering coefficient would contain modular structures in the underlying networks. The modules are a group of proteins interacting with each other and have a higher probability of sharing the same function than two proteins not interacting with each other. The dense sub-networks in a PPI network can, therefore, be identified as functional modules (Ideker and Roded 2008). For example, a study identified chromosomal segregation module consisting of 18 proteins. This complex of 18 proteins is the core of kinetochore and is also found to be responsible for proper alignment and attachment of chromosomes (Chen and Yuan 2006). The interesting revelation of the study indicates that out of these 18 proteins, eight proteins form an interface between kinetochore and microtubule which further promote chromosomal segregation, that are actually two clique structures of size four.

**Table 2:** Random network models

Network science aims to build models that reproduce the properties of real networks. Here, we describe broadly used three random network models.
1. Erdös - Rènyi(ER) network: ER characterizes random graphs and depicts that many of the properties of such networks can be calculated analytically. Construction of an ER random graph with parameter 0≤*p*≤1 and *N* nodes is by connecting every pair of nodes with probability *p*.
2. Small-world (SW) network: This model is characterized by small-world phenomenon of social networks that suggests we are all linked by short chains of acquaintances. Watts and Strogatz SW model is purely built on ER graphs and comprise of properties of high clustering coefficient and short average path (Watts and Strogatz 1998). Friendship networks in social media and gene regulatory networks follow small-world phenomena.
3. Scale-free (SF) network: This model is characterized by an important property of real world networks that most network nodes have a few links to other nodes, however a small number of nodes are highly connected and have a huge number of links to other nodes. This leads to the observation that these networks do not have nodes with a typical number of neighbors, and in this sense these networks are scale-free. Degree distribution of SF follows power law and the power law exponent lies between 2 and 3. Widely used SF generation algorithm is Barabási-Albert (BA) model of preferential attachment (Barabási and Albert 1999). Real-world network such as PPI, transport network follow scale-free behavior (Przulj et al. 2004).

As molecular networks are crucial for cellular information processing and decision making (Karsakov and et al. 2017; Menche and et al. 2015), there are studies performed to further explore topology of molecular networks using graph spectra. The spectra of networks comprising eigenvalues and eigenvectors have been successfully reported to deduce vital inferences when employed to various complex systems including diseases (details in the following sections) (Rai and Jalan 2015; Rai 2017). Following are a few examples where spectra of the networks play an important role in discriminating features of disease networks. In whole-brain functional network analysis, it has been found that patients with autism-spectrum disorders have reduced network clusters as compared to healthy controls which are ultimately involved in a compromised sensorimotor, social, affective and cognitive processing (Sato et al. 2016). In one of the gene co-expression network study, tissues of the breast and ovarian cancers comprised of common cancer-associated modules, and an extend of physiological similarities in two cancers (Zhang 2018). In another gene co-expression network study on severe asthma patients data, researchers characterized immune and non-immune mechanisms and also reported an increased level of T2 inflammation with disease severity (Modena and et al. 2017). Together, network features captured by network spectra can give rise to essential insight into complex diseases.

Furthermore, we broadly discuss the technique used in network spectra. For ease of the readers, we break down list of techniques into three major sections: (I.) eigenvalue distribution of networks, (II.) degenerate eigenvalues, and (III.) eigenvector analysis. The following sections elaborately describe each technique in detail followed by its contribution in understanding complex diseases with relevant examples.

## Network spectra: techniques and applications to system biology of diseases

The spectra (eigenvalues) of the network are known to provide rich information of the topological structure and diffusion of signals. Essentially, this rich information in the underlying system indirectly delivers the blueprint of the complex system. The spectrum of a network is the set of eigenvalues of its adjacency matrix and is denoted as *λ*_*i*_, where *i*=1,2,...,*N* such that *λ*_1_>*λ*_2_≥*λ*_3_≥...≥*λ*_*N*_. In the following, we discuss how the spectral properties helps in unveiling the complexities of the diseases.

### I. Eigenvalue distribution for disease networks

**Fig. 2:**
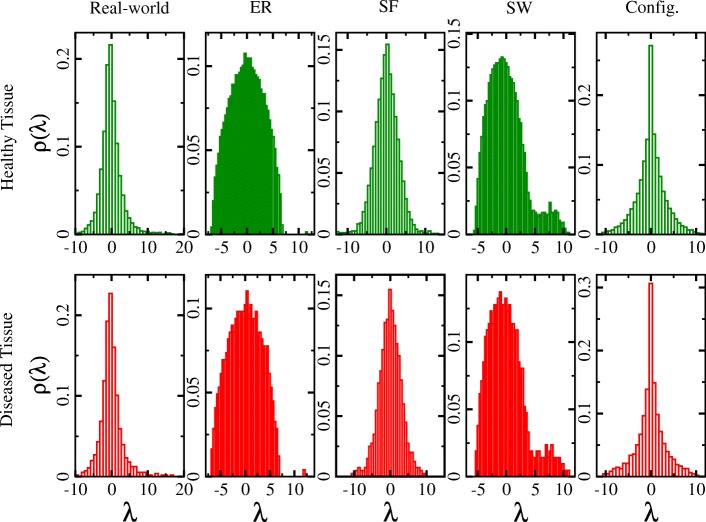
Eigenvalues Distribution. The eigenvalues distribution plotted for healthy (network size (*N*) = 2083, average degree (〈*K*〉) = 10) and the diseased (*N* = 656, 〈*K*〉 = 11) tissues PPI networks of Diabetes Mellitus proteomics data. Also, random networks were constructed using network information corresponding PPI network data. The eigenvalue statistics of PPI reflects typical triangular shape with the tail of the distribution relating with the exponent of the power law of degree distribution as observed for many other biological and real-world networks. ER networks show typical semi-circular shaped distribution. Apart from that SW, SF and configuration model network show different patterns of distribution than their corresponding PPI networks suggesting PPI networks display different behavior than random network

The spectra of a network can be divided into two parts, (i) bulk part consisting of non-degenerative eigenvalues, (ii) extremal and degenerative eigenvalue. The basic investigation of bulk part of eigenvalues is carried out through their density distribution. The spectral density of a graph is the density of the eigenvalues of its adjacency matrix. For a finite system, this can be written as a sum of *δ* functions as,

2 $$ \rho(\lambda) = \frac{1}{N} \sum\limits_{j=1} N \delta (\lambda - \lambda_{j}),   $$

which converges to a continuous function with *N*→*∞*. Spectra of various different network models are found to display different density distributions. The description on types of random network models and networks constructed using biological data are given in Tables 1 and 2. Also, eigenvalue distribution of real-world and random network models is displayed in Fig. 2. For Erdös - Rènyi(ER) network, the spectral density is known to follow a semi-circular distribution (Mehta 1991). This network model assumes that each pair of the graph’s vertices to be connected with equal and independent probabilities, treating a network as an assembly of equivalent units. ER networks have been used for modeling systems made up of large assemblies of similar units. While the semi-circle law is known to describe the spectral density of ER random graphs, much less is known about the eigenvalues of real-world biological graphs. The spectral densities of real-world graphs have specific features depending on the details of the corresponding models. In particular, small-world (SW) and scale-free (SF) network models are largely used for comparing networks constructed using biological data. Small-world network models, created by randomly rewiring some of the edges of a regular ring graph, have a complex spectral density function with many sharp peaks. Various studies using the real data could deduce the similar eigenvalue distributions when compared with modeling SW complex systems (Farkas and et al. 2001; de Aguiar M A M and Bar-YamY 2005; Goh et al. 2001; Dorogovtsev et al. 2003; Palla and Vattay 2006; Bandyopadhyay and Jalan 2007). The SF network model assumes a random graph to be a growing set of vertices and edges, where the location of new edges is determined by a preferential attachment. SF network has triangular shape of density distribution having exponential decay around the center with the tail of the distribution relating with the exponent of the power law of degree distribution on both the sides (Farkas and et al. 2001; de Aguiar M A M and Bar-Yam Y 2005). Figure 2 presents an example of PPI networks of pancreatic healthy cell as well as Diabetes Mellitus II displaying triangular shape of density distribution (Fig. 2). Similar distribution have been observed for PPI networks of various cancers and their normal counterparts in an another study (Shinde and et al. 2015; Rai and et al. 2017). The spectral density of networks pertaining triangular structure depicts a scale-free network topology and a sparsely connected network structure (Rai et al. 2014; Rai and et al. 2017; Agrawal and et al. 2014; Sarkar and Jalan 2016).

Biological networks including disease networks while following triangular density distribution of spectra, encompass some of the distinct features than corresponding model networks *i.e.,* scale-free nature. Few of these distinct features are that real-world biological networks have a very high peak at zero eigenvalue (de Aguiar M A M and Bar-Yam Y 2005). For example, gene co-expression networks of zebra fish and PPI networks of eight biological species manifested triangular density distribution of spectra (Takahashi et al. 2012). These co-expression and PPI networks are commonly used as biological model systems to study human diseases.

Furthermore, apart from density distribution, spacing distribution of eigenvalues have also been analyzed to understand the complexity of disease networks. The spacing distribution of biological and many other real-world networks have shown to follow the universal distribution of random matrices. This behavior of biological networks following those of the random matrices remain to be one of the fascinating discoveries for random matrix community (Bandyopadhyay and Jalan 2007). Using the techniques developed in random matrices, the deviation from universal behavior was further used to understand “randomness" in the underlying network structure (Bandyopadhyay and Jalan 2007; de Aguiar M A M and Bar-Yam Y 2005; Palla and Vattay 2006; Kikkawa 2018). This connection between “randomness" and spectra was used in a study to show that breast cancer PPI networks are more random than the PPI networks of healthy breast tissues (Rai et al. 2014).

### II. Degenerate Eigenvalues identifying local structures in disease networks

As discussed in the previous section, many real-world networks have very high degeneracy at 0 (zero) and sometimes at − 1 (minus one) eigenvalues.

#### Degeneracy at zero (0) eigenvalue

**Fig. 3:**
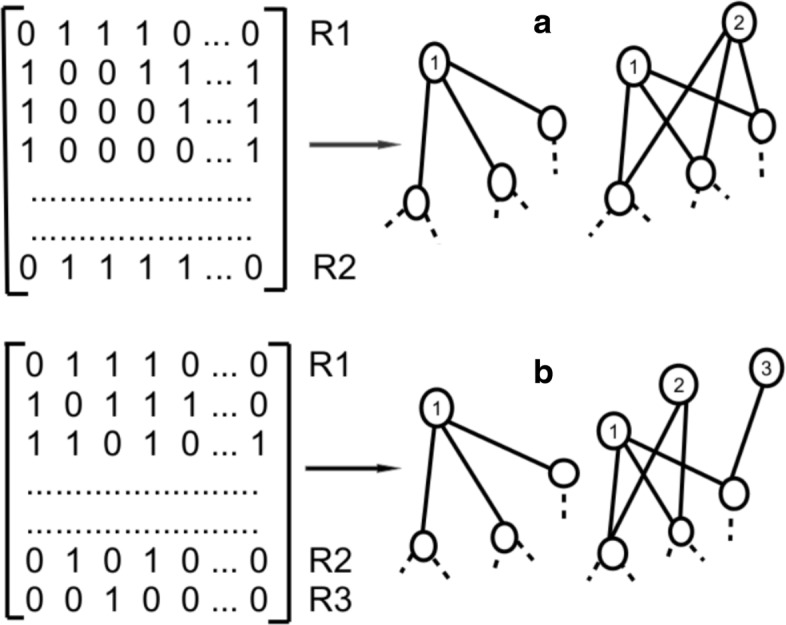
Zero Degeneracy. Schematic diagram representing (**a**) complete node duplication and (**b**) partial node duplication in networks. Biological networks know to posses a higher degeneracy at the zero eigenvalue than corresponding random networks. The degeneracy at the zero eigenvalue is signature of presence of node duplication in the network. The detailed explanation is given in Table 3

The eigenvalue distribution of many real networks, particularly technological and biological networks such as protein-protein interactions of diseases, exhibit high degeneracy at zero eigenvalues (Agrawal and et al. 2014; de Aguiar M A M and Bar-Yam Y 2005; Dorogovtsev et al. 2003). This degeneracy at the zero eigenvalues reveals the evolutionary mechanisms involved in the formation of a complex system (Table 3 and Fig. 3). For PPI networks, gene duplication is one of the reasons behind the occurrence of high degeneracy at zero eigenvalue (Kamp and Christensen 2005). During the cell division and genome replication, occasionally an extra copy of gene get synthesized. Immediately following a duplication event, both the original protein and the new extra copy have the same structure, so both interact with the same set of partners. Consequently, each of the protein partners that interacted with the ancestor gains a new interaction. The gene duplication phenomena plays a key role in the growth, development, evolution and maintenance/stability of biological system (Kamp and Christensen 2005; Teichmann and Babu M. 2004).

**Table 3:** Degeneracy at *λ*_0_ and *λ*_−1_

Mathematically in a network, duplication of nodes yields the same neighbors for two nodes in the corresponding adjacency matrices (Kitano 2004; Yadav and Jalan 2015). It has been shown that duplication of nodes leads to lowering of the rank of the corresponding matrix, hence contributing one additional zero eigenvalue in the spectra (Banerjee and Jost J 2007; Yadav and Jalan 2015; Shinde and et al. 2015). For the adjacency matrix of size *N* and rank *r*, the matrix has exactly *N*−*r* zero eigenvalues (Poole 2006). We discuss here three possible cases when the rank can lower in an adjacency matrix:
(i) two rows (columns) have exactly same entries, it is termed as complete row (column) duplication *R*_1_=*R*_2_,
(ii) the partial duplication of rows (columns) where *R*_1_=*R*_2_+*R*_3_, where, *R*_*i*_ denotes *ith* row of the adjacency matrix (Fig. 3). This condition is computationally exhaustive as to find this state in the matrix has large possibilities (Yadav and Jalan 2015),
(iii) if there is an isolated node in the network, the row and column corresponding to it has zero entries and thus the rank of the matrix is lowered by one. For a connected network, the number of zero eigenvalues (*λ*_0_) provides an exact measure of (i) and (ii) conditions (Yadav and Jalan 2015; Golub and Van-Loan C 2012). Similarly, the calculations for identification of degenerate − 1 eigenvalues is given in (Marrec and Jalan 2017) for A + I matrices.

For example, PPI networks of six different lifestages of *Caenorhabditis elegans* have displayed zero eigenvalue degeneracy (Shinde and Jalan S 2015). Interestingly, PPI network of each lifestage found to have different counts of zero eigenvalues. What important here is, that the genome of an organism remains the same in all the life stages, still there is an occurrence of a different count of duplicate proteins in each lifestage. Similarly, cancer genomes tend to use DNA mutations as agents for clonal duplication and proliferation (Furlong 2013). The Cancer Genome Atlas Pan-Cancer data showed whole-genome doubling determined using somatic copy number alterations (Zack et al. 2013). Essentially, common patterns of somatic copy number alterations were detected across cancer types, including duplication of large region of chromosome (Hsieh and et al. 2013).

In (Rai et al. 2014), authors used empirical data from publically available proteomics databases (UniprotKB and STRING) to construct PPI networks for healthy and cancer breast tissue proteome. Apart from that they compared their real-world networks with corresponding random models such as ER and configuration models. It was found that there exists a very high degeneracy in the real-world biological networks as compared to their corresponding model networks (Shinde et al. 2015; Rai et al. 2014; Rai et al. 2017; Rai et al. 2015). The corresponding configuration models, which generate a network from a given degree sequence, also does not exhibit a high degeneracy at the zero eigenvalue. This observation indicates that, not only a particular degree sequence, but also the nature by which these proteins interact in the network contribute on the occurrence of high degeneracy at the zero eigenvalues in the real networks. Another study related to the PPI networks of normal and cancer oral tissue proteome data reveals that despite similar overall spectral properties (Shinde et al. 2015), the height of the peak at zero eigenvalue differs considerably in both the networks (Table 3 and Fig. 3). Using the direct relation between the zero eigenvalues and the number of duplicate nodes (Shinde et al. 2015), generated a list of nodes participating in the duplication phenomenon. Examining this list, it was revealed that the nodes exhibiting duplication phenomena in healthy tissues were destroyed in the diseased state and additionally new duplicate nodes appear in cancer. This might affect the stability of the system making it more substantial (Bailey et al. 2002) and resistant to drugs (Dean et al. 2005; Gottesman 2002). To summarize, the degeneracy at zero eigenvalue in diseases is shown to arise from the preserved important interactions responsible for the occurrence of the disease and may further lead to failure in the treatments.

#### Degeneracy at -1 (minus one) eigenvalues

Like zero eigenvalue degeneracy, minus one eigenvalue degeneracy has been observed in real-world biological networks including disease networks (Mieghem 2011). Occurrence of zero and minus one eigenvalues in disease networks indicate the presence of complete sub-graphs or cliques (Rai et al. 2017) (Table 3). Cliques are known to be the building blocks of the network and makes a network highly robust and stable (Milo et al. 2002; Yeger-Lotem et al. 2004; Dwivedi and Jalan 2014; Shinde et al. 2018). Presence of large number of complete sub-graphs have been displayed by disease networks. This may be one of the reasons for robustness of the underlying system. Recently, the local structures corresponding to minus one degenerate eigenvalues were identified (Marrec and Jalan 2017). In another multi-cancer PPI network study, (Shinde et al. 2018) identified symmetrical structures in the underlying PPI networks and further picked up proteins forming these essential network structures as candidate proteins. These identified proteins have shown to perform important pathway roles with downstream bioinformatics analysis. Importantly, the identified proteins corresponding to patterns linked to minus one eigenvalue degeneracy did not take any significant structural position in weighted multi-cancer PPI network and hence they were not detectable using various measures such as node degree, clustering coefficient and betweenness centrality.

Overall, the origins of degeneracy at particular eigenvalues are more complex. The study of eigenvalues and their multiplicities is not sufficient to determine the number and size of these structures in networks. It has been recently reported that eigenvectors associated with the degenerate eigenvalues shed light on the structures contributing to the degeneracy (Marrec and Jalan 2017) further illustrating the nodes that contribute to degenerate eigenvalues. The nodes participating in structural modules or patterns leading to degenerate eigenvalue(s) can be best identified by their associate eigenvalues and then can be potential drug targets in treating complex diseases. In addition to eigenvalues, eigenvectors can also be exploited to get information about underlying complexities of the disease states as well as for identifying nodes which might be important for occurrence of a disease. In the following, we discuss the eigenvector analysis performed by the localization property of eigenvectors.

### III. Identification of putative drug targets using the Inverse Participation Ratio (IPR)

Let us understand localization in terms of a localized disease where an infectious process (e.g., cancer spread) that originates in- and is confined to-one area of the organ system. In another example of disease spread, geographical border protection is one of the preventive measure used to control disease epidemic. The aim here is to prevent an infected person to affect other people or to restrict disease to a geographical region (Germann et al. 2006). The IPR is one of the broadly used measure to study the localization of eigenvectors in complex systems such as infectious disease spread, identification of communities in molecular networks etc (Plerou et al. 1999). The mathematical definition of calculating IPR is given in Table 4. Localization depends on the topology of the network and describes the ability to perturbation propagation through the network (Suweis et al. 2015). Few of the recent investigations on eigenvector localization using IPR have revealed the collective influence shown by a set of distinct structural as well as spectral features on the localization properties of principal eigenvector (Goltsev et al. 2012; Pradhan et al. 2017). This phenomena proposed to give some of interesting insights into the spreading processes in the underlying systems.

**Table 4:** Inverse participation ratio (IPR)

**Fig. 4:**
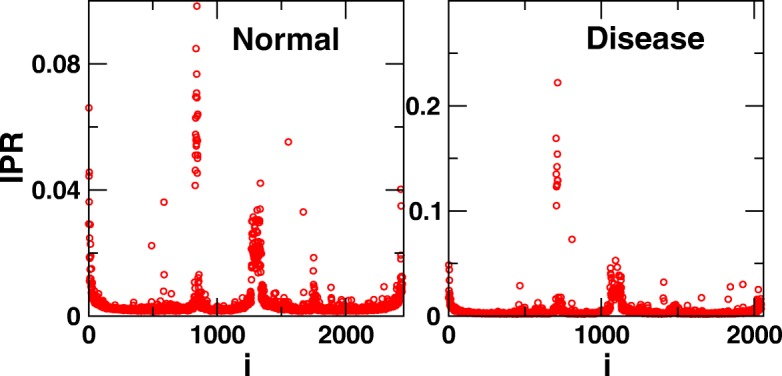
Eigenvector Localization. The figure shows IPR of both disease and normal networks, clearly reflecting three regions (i) degenerate part in the middle, (ii) a large non-degenerate part which follow GOE statistics of RMT and (iii) non-degenerate part at both the end and near to the zero eigenvalues which deviate from RMT (Rai et al. 2014). Moreover, the nodes corresponding to the structures prescribed by the localized eigenvectors can be identified in the networks to further exploit them for the deeper biological understanding

The distribution of eigenvectors components can be used to obtain non-random system dependent information (Fig. 4). The inverse participation ratio (IPR) has long been employed to analyze localization properties of the eigenvectors (Haake and Zyczkowski 1990). For $E_{l}^{k}$ denoting *l*th component of *k*th eigenvector *E*^*k*^, the IPR of an eigenvector can be defined as
$ I^{k} = \frac { \sum _{l=1}^{N} \left [E_{l}^{k}\right ]^{4}}{ \left (\sum _{l=1}^{N} \left [E_{l}^{k}\right ]^{2}\right)^{2}} \text {(3)} $
which shows two limiting values: (i) a vector with identical components $E_{l}^{k} \equiv 1/\sqrt {N}$ has *I*^*k*^=1/*N*, whereas (ii) a vector, with one component $E_{1}^{k}=1$ and the remainders zero, has *I*^*k*^=1. Thus, the IPR quantifies the reciprocal of the number of eigenvector components that contribute significantly.
Further, the average IPR in order to measure an overall localization of the network is calculated as (Jalan and et al. 2011),
$ \langle IPR \rangle = \frac { \sum _{k=1}^{N} \left [I^{k}\right ]}{N} \text {(4)} $
Note that IPR defined as above, separates out the top contributing nodes by keeping the threshold as 1/*IPR*. These Top Contributing Nodes (TCNs) are further found to have important role in the underlying system.

**Fig. 5:**
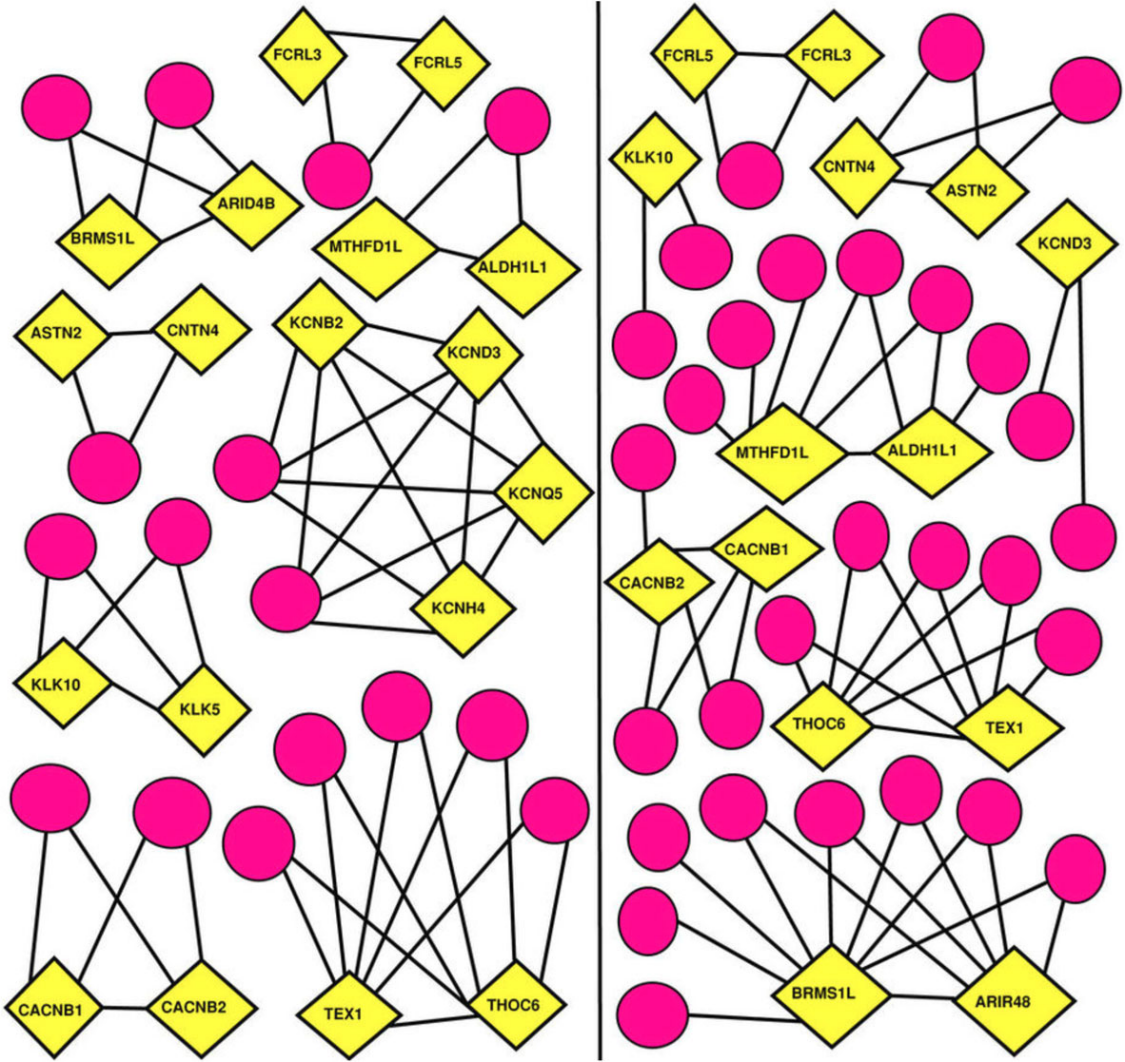
Local structure of top contributing nodes (TCNs). Left panel denotes the local structure of all TCNs in the disease network whereas right panel denotes the local structure for the same proteins in the normal network. Yellow represents TCNs and pink represent their first neighbor. The TCNs, in addition to the functional importance pertaining to the occurrence of the disease state revealed, exhibits interesting structural properties. This is more remarkable in the light that all of these TCNs lie in the low degree regime in the networks. Moreover, their betweenness centrality also are zero further ruling out any trivial structural significance of these nodes. But importance of these nodes based on the analysis of their interactions reveals the existence of preserved local structural patterns. Most strikingly, all of them follow phenomenon of gene duplication which shows TCNs being involved in the pair formation in which first node in each pair has exactly the same neighbors as of the second node. Most remarkably, there are 20 duplicates (proteins having the same number of neighbors and having more than one connection) in the whole network of which 18 are found in the TCNs of the most localized eigenvectors (Rai et al. 2014)

Further, the localized eigenvectors provide information about the top contributing nodes (TCNs) in networks (Fig. 4) *i.e.,* those nodes which contribute the most in the eigenvectors. These nodes can be important for occurrence of disease. Using TCN in the localized eigenvectors, important proteins were detected in Diabetes Mellitus-II (Rai et al. 2015). The proteins corresponding to TCNs in PPI network of Diabetes Mellitus-II proteome were found to be related with insulin resistance and pathways promoting obesity. The TCNs, in addition to the functional importance pertaining to the occurrence of the disease state, may exhibit interesting structural properties (Fig. 4). In a study, such local nodes were found to be a part of clique structures (Fig. 5) displaying the property of gene duplication (Rai et al. 2014). Essentially, the functional importance of these TCNs having gene duplication behavior reveals their involvement in causing the disease and thus proposed as potential drug targets (Rai et al. 2014; Rai et al. 2015). Another study of eigenvector localization on Alzheimer’s disease entails that the TCNs corresponding to the localized eigenvectors have low degree and do not lie in the list of hub proteins depicting a scale-free behavior (Jalan and et al. 2010).

Thus, eigenvectors provide an insight into the important nodes in the disease networks as potential drug targets.

## Conclusion and future scope

Spectral graph theory has been successfully applied to the study of the topology of various disease networks, from the global perspective of their scale-free, small-world nature, to the functionally interacting motifs, symmetrical structures, clusters and the specific interactions between different biomolecules in complex diseases. In disease cells, molecular interactions are different which largely control disease survival and spread. Thus, complex behaviors such as invasion, which are controlled by several specific pathways, are evidently regulated differently than in normal cells (Koutsogiannouli et al. 2013). For example, cell growth and division pathways of metastatic cells in normal cells are terminated after some time, whereas in diseased tissues there is continued execution. Overlapping behaviors among normal and disease cells shown by various spectral tools such as triangular structure of density distribution, high degeneracies at zero and minus one eigenvalues, suggest that though organization of metabolic and signaling networks is differently regulated in the two cell types, there are large amount of similarities in the complexities of the pathways organization where pathway-agents might be different. It remains mostly speculative how different cells execute complex final functions (proliferation, spread, invasion, etc.) having conserved pathway structures and with the help of the identical primary genome sequence (Koutsogiannouli et al. 2013; Rai et al. 2014). Nodes and pathways identified using information of degeneracies at zero and minus one eigenvalues and IPR provide essential sub-graphs or set of nodes for drug-target. In a way, RMT presents new practical tools for identification of pathway agents (genes, proteins, etc.) responsible for the occurrence of the disease as well as provide insight into the complexity of the disease at the rudimentary level.

Overall, spectral graph theory framework has helped in uncovering the complexity at the fundamental level enabling us to have a global view of the diseasome. However, the understanding of the biological phenomenon in disease networks using graph spectra is still at the budding stage. The studies using graph spectra can help in improving our current knowledge of molecular associations in disease models in a time-efficient and cost-effective manner. Employing such a technique has already shown its promise lead to further advancements in disease diagnosis, prognosis, and identification of novel drug targets for disease therapy. This novel approach provides a clue to developing the promising and nascent concept of single drug therapy for multiple diseases, biomarkers useful in disease diagnosis as well as personalized medicine. The holistic framework of networks together with the spectral analysis may be useful for diseases wherein the knowledge of the abnormal gene/protein(s) function(s) is unavailable.

## Abbreviations

APID: Agile protein interactomes dataServer
BA model: Barabási-albert model
BioGRID: Biological general repository for in- teraction datasets
BIGG: The biochemical genetic and genomics knowledge base
BIND: The biomolecular interaction network database
ChIP: Chromatin immuno-precipitation
ChIP-seq: Chromatin immuno-precipitation sequencing
DIP: The database of interacting proteins
DM-II: Diabetes mellitus-II
ER: Erdös - rènyi
HIPPIE: Human integrated protein-protein interaction reference
HMDB: The human metabolome database
HPRD: The human protein reference database
IMEX: Inter- national molecular exchange
IntAct: The protein inter- action database
IPR: Inverse participation ratio
KEGG: Kyoto encyclopedia of genes and genomes
MINT: The molecular interaction database
MIPS: Munich information center for protein sequence
PPI: Protein-protein interaction
RMT: Random matrix theory
SF: Scale-free
SW: Small-world
TCNs: Top contributing nodes
